# Validation of the Nassar Preoperative Risk Scale to Predict Intraoperative Difficulty in Patients Undergoing Laparoscopic Cholecystectomy

**DOI:** 10.7759/cureus.116880

**Published:** 2026-09-24

**Authors:** Arunav Roy, Nabu Kumar, Reema Bhagat, Dipendra K Sinha, Anmol Kujur, Md Istamaul Khan, Pragya Sinha, Mrinank Harshvardhan, Sumedha Ghosh, Swetav Suman

**Affiliations:** 1 Plastic Surgery, Rajendra Institute of Medical Sciences, Ranchi, IND; 2 General Surgery, Rajendra Institute of Medical Sciences, Ranchi, IND

**Keywords:** difficult cholecystectomy, laparoscopic cholecystectomy, nassar score, preoperative risk stratification, surgical prediction

## Abstract

Background

Laparoscopic cholecystectomy (LC) is one of the most commonly performed surgical procedures worldwide. Although generally safe, a subset of procedures may become technically challenging due to various intraoperative factors. Difficult LC is associated with increased operative time, higher complication rates, and greater risk of bile duct injury. Preoperative prediction of operative difficulty may allow better surgical planning and allocation of experienced surgeons. The Nassar Preoperative Risk Scale has been proposed as a tool to predict difficulty in LC; however, validation in different populations remains limited. This study aimed to evaluate the predictive accuracy of the Nassar Preoperative Risk Scale in predicting difficult LC.

Methods

A hospital-based analytical cross-sectional study was conducted from November 2024 to March 2025 among patients undergoing LC. Consecutive patients aged 18-80 years with calculous cholecystitis undergoing LC were included. The Nassar Preoperative Risk Scale was calculated for each patient. Intraoperative difficulty was assessed using the Nassar intraoperative difficulty grade. Receiver operating characteristic (ROC) analysis was performed to determine the optimal cut-off value and evaluate the predictive performance of the scale.

Results

A total of 572 patients were included in the study. Easy intraoperative findings were observed in 87.24% of cases, while 12.76% were classified as difficult. ROC analysis demonstrated good predictive performance of the Nassar Preoperative Risk Scale, with an area under the curve (AUC) of 0.884. The sensitivity was 76.7%, specificity was 87.8%, and overall predictive accuracy of the scale was 86.4%. The positive predictive value (PPV) was 47.86%, and the negative predictive value (NPV) was 96.26%.

Conclusions

The Nassar Preoperative Risk Scale demonstrated good predictive accuracy in identifying difficult LC. Preoperative risk stratification using this scoring system may help surgeons anticipate operative challenges, improve surgical planning, and enhance patient safety.

## Introduction

Laparoscopic cholecystectomy (LC) is currently one of the most commonly performed surgical procedures worldwide [1]. While LC is generally considered a safe and routinely performed procedure, various intraoperative and patient-related factors can make the procedure challenging. Difficult LC, as defined by Maehira et al., refers to procedures in which various factors complicate the safe and timely completion of surgery [2]. It poses various challenges and is seen to have higher complication rates compared to a routine LC, including accidental injury to vital structures, bile leaks, peritonitis, and life-threatening infections. These necessitate additional surgeries, prolonged hospital stays, long-term morbidity, or mortality for patients [3,4].

The fraction of difficult LCs is around 15% [3]. Various studies have found that the mortality rate in difficult LC is much higher than in routine LC. In a study by Abdallah et al., the mortality rate was four times higher in difficult LC than in routine LC patients, while Coccolini et al. reported a 20% increased risk of mortality and double the risk of postoperative morbidity in difficult LC [5-9].

Difficulty is a subjective variable, and various studies have attempted to objectify difficulty in LC. One of these is the Parkland grading scale introduced by Madni et al. [10], which focused on quantifying difficulty grading based on intraoperative adhesions, while Griffiths et al. published their difficulty grade based on various intraoperative presentations of the gallbladder (GB), cystic pedicle, and adhesions encountered intraoperatively [11].

Just quantifying difficulty is not enough; it would be a blessing for the surgeon and the patient to be able to predict which LC will be difficult in advance. This will help to triage patients who have a higher probability of difficult LC so that these cases may be assigned to senior or more experienced surgeons. This prior knowledge will also assist surgeons in planning their approach accordingly, reducing the likelihood of adverse outcomes.

To address this challenge, various preoperative risk scoring systems have been developed to predict intraoperative difficulty and guide decision-making [12-15]. Among the various preoperative scoring systems, the Nassar Preoperative Difficulty Scale is one such score that stands out as being accurate and valid [16]. The Nassar scale uses several key variables, such as age, sex, American Society of Anesthesiologists (ASA) grade, diagnosis at admission, GB wall thickness, common bile duct (CBD) dilatation, endoscopic retrograde cholangiopancreatography (ERCP) status, and type of admission [16,17].

Even though it is widely accepted, the Nassar score may have variable performance in different populations. Only a handful of studies have been published validating the Nassar scoring system in the Indian population [18-20]. All of these studies are either from the southern or western part of India. Validation of this scoring system in our population from eastern India will shed better light on the application of the Nassar score in different population groups.

We hypothesized that the Nassar Preoperative Risk Scale would demonstrate good predictive accuracy for identifying difficult LC in our population. We thus conducted this study with the aim of determining the predictive accuracy of the Nassar Preoperative Risk Scale for predicting intraoperative difficulty correctly in patients undergoing LC, with the primary objective of determining the sensitivity, specificity, positive predictive value (PPV), negative predictive value (NPV), and area under the curve (AUC) of the Nassar Preoperative Risk Scale for predicting difficult LC.

## Materials and methods

This study was reported in accordance with the Standards for Reporting Diagnostic Accuracy Studies (STARD) guidelines [21].

Study design

We used a hospital-based analytical cross-sectional study design (predictive accuracy test) to compare the Nassar Preoperative Risk Scale with intraoperative difficulty in LC. The study was conducted from November 2024 to March 2025.

Participants

All consecutive patients with calculous cholecystitis planned for LC at a tertiary care teaching hospital in Eastern India were included, irrespective of sex, between 18 and 80 years of age. LCs performed by surgeons with experience of fewer than 150 LC surgeries were excluded from the study [22].

Of 589 participants assessed for eligibility, nine were excluded as they did not fulfill the inclusion criteria, mainly due to the age cut-off. Among the remaining 580 participants, eight declined consent; hence, 572 were enrolled in the study (Figure 1).

**Figure 1:**
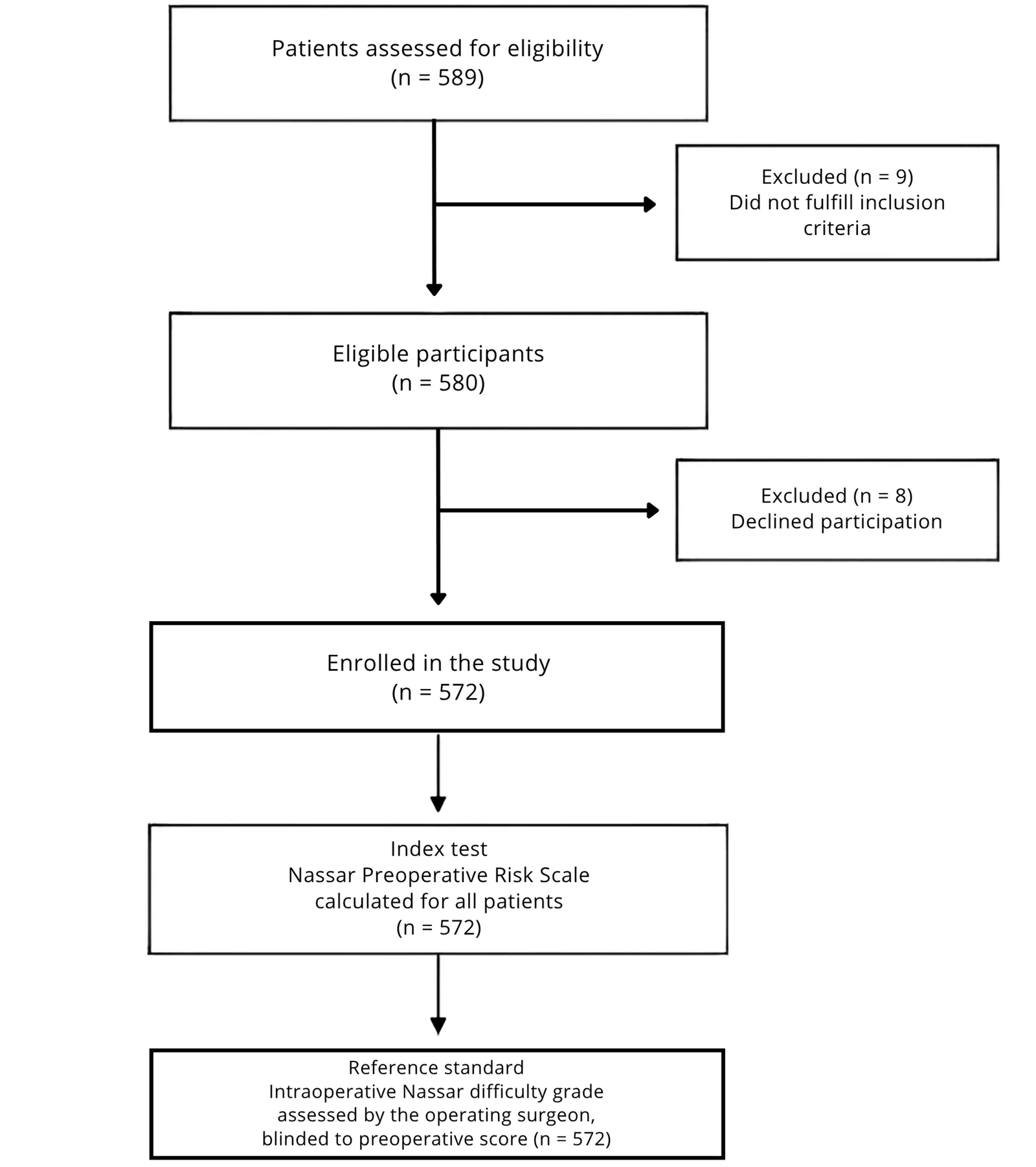
Participant Allotment (STARD Flow Diagram) STARD: Standards for Reporting Diagnostic Accuracy Studies

Study size

A study on cholelithiasis by Siddiqui et al. [23,24] documented a prevalence of 38%. Assuming sensitivity and specificity of 94% and 86%, respectively, and a confidence level of 95% with 10% loss to follow-up, a required sample size of 570 was calculated.

Variables

After obtaining informed consent from patients, the Nassar Preoperative Risk Scale was calculated. This included age as a continuous variable, and gender, ASA classification, primary diagnosis, GB wall thickness (≥3 mm), CBD dilatation (>6 mm), preoperative ERCP, and type of admission as categorical variables (Table 1). The minimum score of the preoperative scale is 0, and the maximum score is 19.

**Table 1:** Preoperative Risk Scale for Difficult Laparoscopic Cholecystectomy ASA: American Society of Anesthesiologists; CBD: common bile duct; ERCP: endoscopic retrograde cholangiopancreatography Low risk: 0-1; intermediate risk: 2-6; high risk: 7-19. Adapted with permission from Nassar et al. [16]

Variable	Points
Age < 40 years	0
Age ≥ 40 years	1
Sex: female	0
Sex: male	1
ASA grade 1	0
ASA grade 2	1
ASA grade 3	2
ASA grade 4	7
Diagnosis: pancreatitis	0
Diagnosis: biliary colic	0
Diagnosis: choledocholithiasis	1
Diagnosis: cholecystitis	4
Thick-walled gallbladder (≥3 mm): no	0
Thick-walled gallbladder (≥3 mm): yes	2
CBD dilation (>6 mm): no	0
CBD dilation (>6 mm): yes	1
Preoperative ERCP: no	0
Preoperative ERCP: yes	1
Admission: elective	0
Admission: delayed	1
Admission: emergency	2

Intraoperative difficulty was assessed using the Nassar Intraoperative Difficulty Scale for LC (Table 2), where grades 1 and 2 were labeled as easy and grades 3 and 4 as difficult. Intraoperative difficulty was assessed by the operating surgeon, who was blinded to the patient's preoperative Nassar score, the criteria of which were defined a priori and applied uniformly across all operating surgeons in the unit. As grading was performed prospectively by the single operating surgeon for each patient rather than by multiple independent assessors of the same procedure, formal interobserver agreement was not assessed. Intraoperative difficulty was recorded as the outcome variable.

**Table 2:** Intraoperative Difficulty Scale for Laparoscopic Cholecystectomy Easy: grades 1-2; difficult: grades 3-4. Adapted with permission from Nassar et al. [17]

Grade	Gallbladder	Cystic pedicle	Adhesions
1	Floppy, non-adherent	Thin and clear	Simple up to the neck/Hartmann's pouch
2	Mucocele, packed with stones	Fat laden	Simple up to the body
3	Deep fossa, acute cholecystitis, contracted, fibrosis, Hartmann's adherent to common bile duct, impaction	Abnormal anatomy or cystic duct - short, dilated, or obscured	Dense up to fundus; involving hepatic flexure or duodenum
4	Completely obscured, empyema, gangrene, mass	Impossible to clarify	Dense, fibrosis, wrapping the gallbladder, duodenum, or hepatic flexure difficult to separate

Age was estimated as a continuous variable and expressed as mean ± SD. GB wall thickness, CBD dilatation, gender, ASA classification, primary diagnosis, preoperative ERCP, and type of admission were estimated as categorical variables and expressed as proportions. The Nassar Preoperative Scale was estimated as a continuous variable and recorded as the input variable. Intraoperative difficulty was estimated as a categorical variable and recorded as the outcome variable.

Statistical methods

Descriptive analysis of all variables was performed. Binomial logistic regression was performed using the Nassar Preoperative Risk Scale as the independent variable and intraoperative difficulty as the dependent variable to assess the association between the score and operative difficulty. A receiver operating characteristic (ROC) curve was plotted, and the AUC was calculated to quantify overall discrimination across all possible thresholds. The optimal cut-off value was determined separately as the score maximizing Youden's index (sensitivity + specificity − 1).

Sensitivity, specificity, PPV, NPV, and AUC of the preoperative scale in predicting intraoperative difficulty were calculated. Jamovi version 2.6.24 (jamovi project, Sydney, Australia) was used for statistical calculations.

## Results

Age of enrolled patients ranged from 18 to 79 years, with a mean age of 46.03 years. We observed a female predominance, with a male-to-female ratio of 1:2.48. The most common ASA grade was grade 2, with 73% (n = 418) of participants categorized in this group. The next most common ASA grade was grade 1, with 19.7% (n = 113), followed by grade 3, with 7.1% (n = 41) (Table 3).

**Table 3:** Characteristics of the Study Base at Enrollment (n = 572) ASA: American Society of Anesthesiologists; CBD: common bile duct; ERCP: endoscopic retrograde cholangiopancreatography

Variable	n
Age < 40 years	149
Age ≥ 40 years	423
Male	164
Female	408
ASA grade 1	113
ASA grade 2	418
ASA grade 3	41
ASA grade 4	0
Diagnosis: pancreatitis	32
Diagnosis: biliary colic	0
Diagnosis: cholecystitis	540
Diagnosis: choledocholithiasis	0
Thick-walled gallbladder (≥3 mm): no	484
Thick-walled gallbladder (≥3 mm): yes	88
CBD dilation (>6 mm)	0 (none observed)
Preoperative ERCP	0 (none observed)
Admission: elective	540
Admission: delayed	32
Admission: emergency	0

In our study, 5.59% (n = 32) of cases had concomitant pancreatitis. The majority of patients underwent elective surgery (94.4%, n = 540), with a small proportion experiencing a delay of at least seven days after the onset of symptoms (5.9%, n = 32).

The Nassar Preoperative Risk Scale ranged from 1 to 11 in our study population, with a mean of 6.29 ± 1.65 (Table 4).

**Table 4:** Distribution of Nassar Preoperative Risk Scale Scores Data are presented as number (percentage). Percentages were calculated based on the total study population (n = 572).

Score	n (%)
1	5 (0.87%)
2	9 (1.57%)
3	9 (1.57%)
4	32 (5.59%)
5	102 (17.83%)
6	187 (32.69%)
7	111 (19.41%)
8	60 (10.49%)
9	40 (6.99%)
10	11 (1.92%)
11	6 (1.05%)

The Nassar Intraoperative Difficulty Scale showed that the majority of the study population had easy intraoperative findings, 87.24% (n = 499) (Table 5).

**Table 5:** Characteristics of Intraoperative Difficulty During LC LC: laparoscopic cholecystectomy

Category	n (%)
Easy	499 (87.24%)
Difficult	73 (12.76%)

Binomial logistic regression showed that the Nassar Preoperative Risk Scale was a significant predictor of intraoperative difficulty. Each one-point increase in the score was associated with a 3.48-fold increase in the odds of a difficult procedure (odds ratio 3.48, 95% CI 2.70-4.49; p < 0.001; McFadden R² = 0.368).

ROC analysis for different cut-off values of the Nassar Preoperative Risk Scale showed that the most accurate cut-off value was 8, with a sensitivity of 76.7%, specificity of 87.8%, and AUC of 0.884 (Table 6, Figure 2). The PPV was 47.86%, and the NPV was 96.26%. The estimated precision was 96%, recall was 84%, and overall accuracy was 86.4% (Table 7).

**Table 6:** ROC Cutpoint Analysis for the Nassar Preoperative Risk Scale ROC: receiver operating characteristic; PPV: positive predictive value; NPV: negative predictive value; AUC: area under the curve Cut-off 8 gave the highest combined sensitivity and specificity and was used as the study's operating threshold.

Cutpoint	Sensitivity	Specificity	PPV	NPV	Youden's index	AUC
1	100%	0%	12.76%	N/A	0.0000	0.884
2	100%	1%	12.87%	100%	0.0100	0.884
3	100%	2.81%	13.08%	100%	0.0281	0.884
4	100%	4.61%	13.30%	100%	0.0461	0.884
5	100%	11.02%	14.12%	100%	0.1102	0.884
6	97.26%	31.06%	17.11%	98.73%	0.2832	0.884
7	90.41%	67.54%	28.95%	97.97%	0.5795	0.884
8	76.71%	87.78%	47.86%	96.26%	0.6449	0.884
9	49.32%	95.79%	63.16%	92.82%	0.4511	0.884
10	21.92%	99.80%	94.12%	89.73%	0.2172	0.884
11	8.22%	100%	100%	88.16%	0.0822	0.884

**Figure 2:**
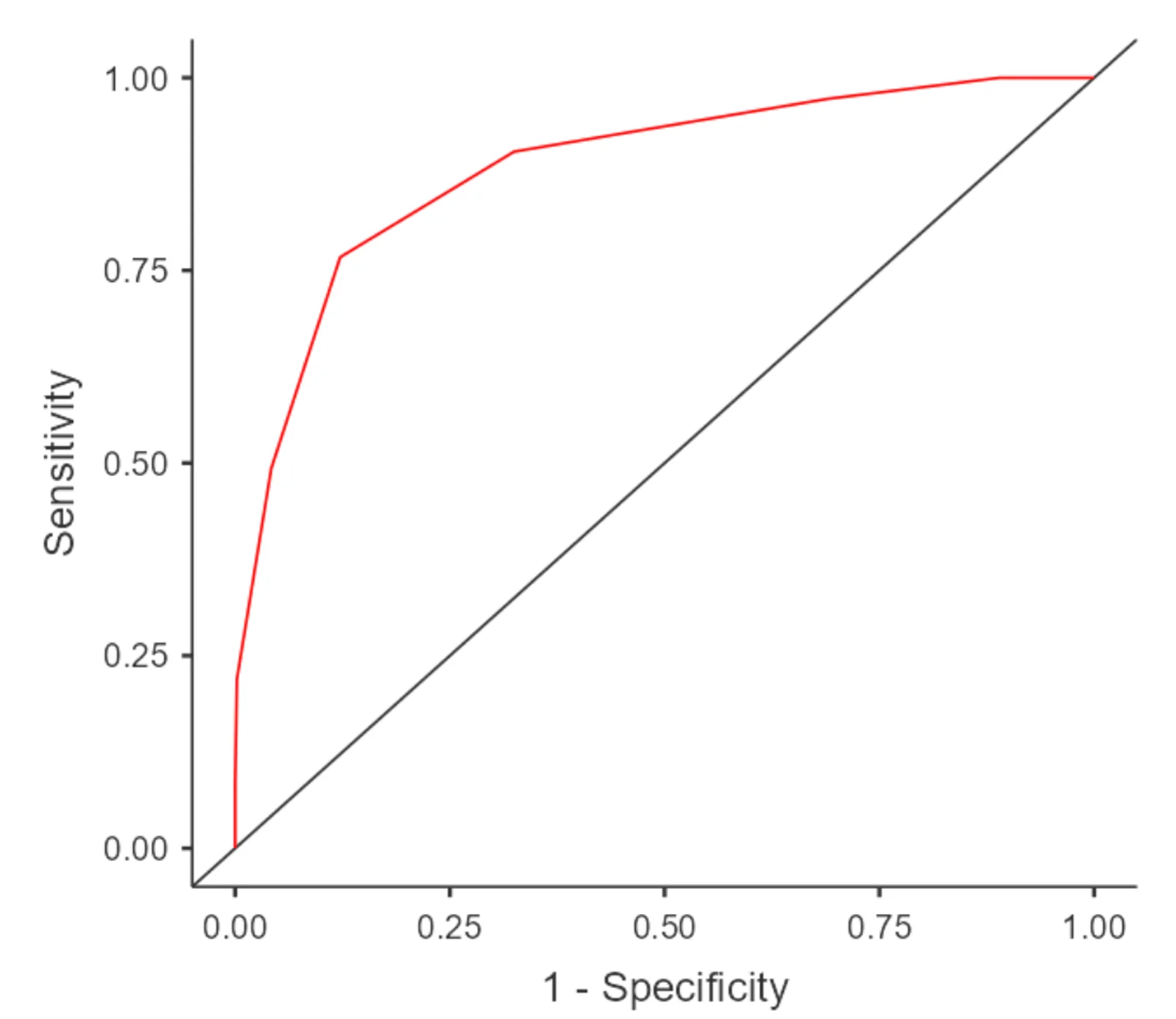
ROC Curve

**Table 7:** Predictive Measures at the Selected Cut-Off AUC: area under the curve Cut-off value set to 0.225 predicted probability (equivalent to a Nassar score of 8).

Accuracy	Specificity	Sensitivity	AUC
0.864	0.878	0.767	0.884

## Discussion

The present study aimed to analyze the predictive capability of the Nassar Preoperative Risk Scale in assessing intraoperative difficulty in LC. The strength of this study lies in its large sample size and the use of a validated intraoperative difficulty grading system to objectively assess surgical difficulty.

Our findings demonstrate that the Nassar scale shows good discriminative ability in predicting difficult LC, with an area under the ROC curve of 0.884. Using a cut-off value of 8, the high sensitivity, specificity, and accuracy suggest that the Nassar Preoperative Risk Scale can serve as a reliable tool for preoperative risk stratification in patients undergoing LC.

Accurate prediction of operative difficulty is important, as difficult LC is associated with higher rates of bile duct injury, increased operative time, conversion to open surgery, and postoperative morbidity [8,9]. Preoperative identification of such cases allows better operative planning and improved preparedness for intraoperative challenges [13]. In this context, the Nassar scoring system provides surgeons with valuable guidance during preoperative assessment [16].

Our findings are consistent with previously published studies evaluating the Nassar scoring system. Nassar et al., who originally developed the scale, reported that the score demonstrated good predictive capability for difficult LC across multiple centers [16]. Subsequent validation studies have also demonstrated satisfactory predictive performance [12,18-20]. The present study adds further evidence in support of the Nassar scale, particularly within the Indian population, where relatively few validation studies have been conducted [18-20].

An important observation in our study is the high NPV (96.26%) of the scale, suggesting that patients with low preoperative risk scores are highly likely to undergo uncomplicated LC. In contrast, the PPV at this cut-off was considerably lower (47.86%). This asymmetry is expected given the relatively low prevalence of difficult LC in our cohort (12.76%), as both predictive values are influenced by outcome prevalence. In practical terms, the scale at the selected threshold is more dependable for identifying patients unlikely to have a difficult procedure than for confidently identifying those who will, since a substantial proportion of patients with high scores ultimately underwent an uncomplicated operation. The scale is therefore best positioned to reassure and streamline low-risk cases, while a high score should prompt heightened preparedness rather than be read as a definitive prediction of difficulty [16,22].

Despite the encouraging findings, certain limitations should be acknowledged. First, the study was conducted at a single institution, which may limit the generalizability of the findings. Second, cases performed by surgeons with fewer than 150 prior LC procedures were excluded; because a central purpose of a preoperative difficulty score is to help triage cases toward more experienced surgeons, the scale's performance in the hands of less experienced operators was not tested in this study [21]. A multicenter study with a larger, more diverse population would be beneficial to validate these results. Additionally, this study focused primarily on preoperative and intraoperative factors; future research could explore postoperative outcomes, including complication rates, length of hospital stay, and patient recovery metrics, to assess the broader impact of preoperative risk assessment.

Some variables included in the Nassar Risk Scale, such as dilated CBD and prior ERCP, were not observed in our study population, which may have influenced the distribution of scores and potentially affected predictive performance. Additionally, the absence of emergency admissions and biliary colic as a primary diagnosis in the study population may also suggest the same.

Furthermore, the optimal cut-off was both selected and evaluated within the same cohort using Youden's index, without a separate internal-validation procedure such as bootstrapping or cross-validation. This may yield optimistic estimates of the cut-off's diagnostic performance. Accordingly, the present findings should be regarded as demonstrating the discriminative performance of the scale in our population rather than as a fully independent validation of the selected threshold, and external validation in an independent cohort remains an important next step.

Nevertheless, the strengths of this study include the relatively large sample size and the use of a standardized intraoperative grading system for assessing operative difficulty. These factors contribute to a more objective evaluation of the predictive accuracy of the Nassar scale.

## Conclusions

In this single-center cohort, the Nassar Preoperative Risk Scale demonstrated good discriminative performance for predicting intraoperative difficulty in LC, with a high NPV at the selected cut-off. These findings indicate that the scale can reliably identify patients unlikely to have a difficult procedure and may thereby inform preoperative planning and case allocation. However, this study evaluated predictive performance only; whether incorporating the scale into practice improves surgical decision-making, alters operative management, or reduces postoperative complications was not assessed and would require prospective, ideally multicenter, evaluation.
